# RiCoRecA: *ri*ch *co*oking *rec*ipe *a*nnotation schema

**DOI:** 10.3389/frai.2025.1550604

**Published:** 2026-01-12

**Authors:** Filippos Ventirozos, Mauricio Jacobo-Romero, Haifa Alrdahi, Sarah Clinch, Riza Batista-Navarro

**Affiliations:** 1 Department of Computing and Mathematics, Manchester Metropolitan University, Manchester, United Kingdom; 2 Department of Computer Science, School of Engineering, University of Manchester, Manchester, United Kingdom

**Keywords:** information extraction, workflow extraction, generative encoder-decoder models, instructional text, language resources, internet of things

## Abstract

Despite recent advancements, modern kitchens, at best, have one or more isolated (non-communicating) “smart” devices. The vision of having a fully-fledged ambient kitchen where devices know what to do and when has yet to be realized. To address this, we present RiCoRecA, a novel schema for parsing cooking recipes into a workflow representation suitable for automation, a step toward that direction. Methodologically, the schema requires a number of information extraction tasks, i.e., annotating named entities, identifying relations between them, coreference resolution, and entity tracking. RiCoRecA differs from previously reported approaches in that it learns these different information extraction tasks using one joint model. We also provide a dataset containing annotations that follow this schema. Furthermore, we compared two transformer-based models for parsing recipes into workflows, namely, PEGASUS-X and LongT5. Our results demonstrate that PEGASUS-X surpassed LongT5 on all of the annotation tasks. Specifically, PEGASUS-X surpassed LongT5 by 39% in terms of F-Score when averaging the performance on all the tasks; it demonstrated almost human-like performance.

## 1 Introduction

In the last few years, we have seen the emergence of smart devices for use within the kitchen. Indeed, devices designed for the Internet of Things (IoT) or the Internet of Robotic Things (IoRT)^1^ that automate cooking tasks in commercial settings are increasingly becoming popular (Bosch, 2023; Thermomix, 2023; Omni, 2023; Xiaomi, 2023). These devices are designed to facilitate cooking processes by offering a variety of automated functionalities. However, their operation is largely isolated, with each device functioning as a standalone unit. Moreover, these devices utilize coded recipes that have undergone manual curation. Cooking instructions, originally in English, Chinese and other languages, are transformed into code that is comprehensible to the specific appliance. Therefore, while these devices can execute automated tasks, they are unable to independently interpret and apply recipes provided by the end-user in their natural language form. Furthermore, the capability of these devices to work with multiple other devices and collaborate seamlessly with the end-user still remains an unrealised objective.

Weiser's vision of Ambient Intelligence (Weiser, 1999) points toward an ideal where ambient intelligence can exist in the background and proactively meet its end-user demands. Bringing this perspective to the kitchen, IoT devices could interpret a cooking recipe provided by the user, create a plan of action and engage in collaborative behavior. However, the actualisation of such systems, where IoT devices and humans work together smoothly, remains a challenging prospect.

We propose the development of natural language processing (NLP) methods for analyzing recipes as a step toward realizing the above-described vision. Given that recipes are a resource that contains instructions that can be used to coordinate different IoT devices (Paternò and Santoro, 2017), our overarching aim is to develop an NLP-based framework for transforming natural language instructions into IoT workflows. The core principle is based on the automatic generation of a workflow from a given recipe, which can then be executed by IoT devices.

We define a workflow as a series of steps—which may or may not be dependent on each other—that are required to complete a task (Ventirozos et al., 2021). IoT functionalities are dichotomised on a granular level into sensors and actuators. The sensors indicate the ability to get a measurement from the real world (e.g., temperature, occupancy), and the actuators interact with the world; they act in some way (e.g., raise temperature, move an object). This is a key concept that underpins how we model a recipe, as discussed in the succeeding sections of this paper.

One can view recipe instructions as a series of functions. For example, the recipe instructions shown on the left-hand side of Table 1, can be formally expressed as the numbered functions on the right-hand side. All of the numbered lines convey actions (actuator-related), except for Lines 5, 8 and 10, which are conditions (sensor-related) that use a code statement (i.e., “Until,” “For,” “If”) and dictate the execution of the action in Line 6 (“stir”), Line 9 (“bake”), and Line 11 (“turn”), respectively. The values enclosed in brackets denote lexical units such as the names of tools, ingredients, or other recipe-related tags. It is worth noting that most of the instructions are dependent on the preceding steps being completed. For instance, Line 4 depends on Line 3; one has to add the ingredients to mix them. However, some instructions may have a long-distance dependency, such as Line 9 needing the oven to be preheated in Line 0.

**Table 1:** The table presents a baked potato recipe in the left column, with its pseudocode representation in the right column.

**Cooking recipe**	**Expressed as pseudo-functions**
Preheat oven to 200 °C (400 °F). Clean and cut the potatoes into similar-sized pieces. In a large bowl, mix olive oil, salt, pepper, herbs, and minced garlic. Add potatoes and stir until they're coated. Place the potatoes on a baking tray. Bake in the oven for about 40–50 min, until they're golden brown and crisp. Turn them over halfway. Serve and Enjoy!	0 | preheat (tool = oven, sett = 200 C, ...) 1 | clean (ingr = potatoes, ...) 2 | cut (ingr = potatoes, sett = smaller-sized...) 3 | mix (tool = large bowl, ingr = [olive oil, salt, ...) 4 | add (ingr = potatoes, ...) 5 | Until (coated, ...): 6 | stir (ingr = [potatoes, olive oil, pepper, ], ...) 7 | place (tool = baking_tray, ingr = potatoes, ...) 8 | For (40–50 min) OR Until (golden brown, ...): 9 | bake (tool = oven, ingr = [potatoes, olive oil, salt, pepper, ...) 10| If (halfway): 11| turn (ingr = [potatoes, olive oil, pepper...) 12| serve (ingr = [potatoes, olive oil, pepper ...])

Here, the main verbs, including actions and conditions, serve as functions, accepting various parameters enclosed in brackets. To maintain brevity, not all parameters and details are included.

Thus, recipe instructions can be expressed as a workflow that can be represented as a graph consisting of nodes and edges, where nodes represent the corresponding functions and edges represent the dependency linkages between these functions. Specifically, we defined a cooking workflow as an encapsulation of the following information: the actions and conditions (which we will refer to as *predicates*), the lexical units involved in each (i.e., entities and their attributes), and the sequence in which the actions should be carried out (e.g., which action depends on which).

Lastly, the ingredients during a cooking process are often referred to differently or are skipped altogether, as they are obvious to the reader of the recipe. For instance, in Line 9, we need to infer the entities that require processing even though they are not explicitly mentioned in the cooking recipe.

Therefore, a number of information extraction tasks need to be applied to any given recipe: named entity recognition, relation classification, coreference resolution and entity tracking. Below, we introduce each of these tasks.

Named entity recognition (NER) is a sub-task of information extraction that seeks to locate and classify named entities (NEs) in a text into predefined categories such as names, organizations, locations, time expressions, quantities, monetary values and so forth. In our case, in the sentence “Preheat oven to 200 °C (400 °F)” our NER system would identify “Preheat” as an action (predicate), “oven” as a tool, and “200 °C (400 °F)” as a device setting.

Relation classification (RC) deals with predicting the semantic relationship between pairs of entities in a text. In our case, the entities correspond to the above NE spans. For example, in Table 1 in the right column, RC would recognize the dependency relationship between “clean” and “cut” as well as “preheat” and “bake,” and that the latter depends on the former. Moreover, it would recognize the lexical units that belong to a predicate. For instance, in the second line on the left pane, the RC would indicate that the “similar-sized pieces” setting belongs solely to the “cut” predicate, but not to “clean.”

Coreference resolution, on the other hand, is the information extraction task of identifying coreferring expressions, i.e., expressions in a text that refer to the same entity or event. This is crucial for understanding the full context of a text, as pronouns (such as “it” or “them”) and noun phrases (such as “dough”) frequently refer back to previously mentioned entities. For example, in Table 1, in the penultimate sentence of the left pane, “them” refers not only to the potatoes but also to other ingredients mixed with the potatoes (i.e., olive oil, salt, pepper, herbs, and minced garlic). Moreover, we extend our task to situations where no pronoun is used but the elements are implied to be involved in a process; we still predict their involvement. We refer to coreference resolution and this extended aspect of coreference resolution as entity tracking, since this process allows us to track the entities in each cooking step. In the remainder of this paper, we use these terms interchangeably.

What makes our work different from previous approaches to automatic workflow construction based on recipes (also referred to in the literature as flowgraph construction [Yamakata et al. 2020]), is our handling of code statements. These code statements allow us to identify actions that are conditioned on some information from the environment that can be determined using *sensing*, e.g., the baking action in “bake until golden brown.” Such predicates are different from immediately executable actions that require *actuation*, e.g., “heat the oven to 400 deg F.” In addition, we utilize notations to determine which processes can be done sequentially, in parallel, repeated or disjunctive (i.e. XOR). To the best of our knowledge, our work is the first to incorporate such information in workflows extracted from text. Crucially, our approach is novel in that it learns these different information extraction tasks jointly within a single sequence-to-sequence model, a significant departure from prior pipeline-based methods that handle these tasks in isolation.

**Figure 1:**
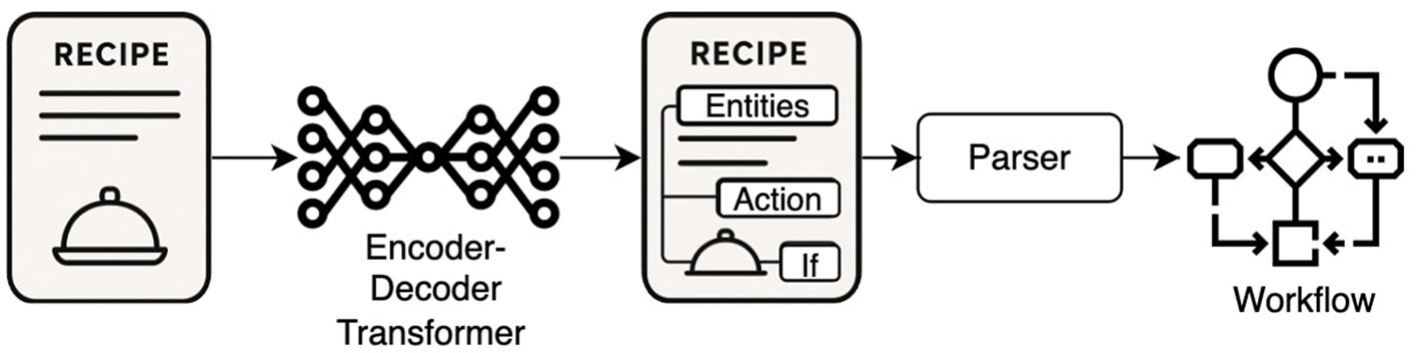
The figure above demonstrates the inference processing pipeline. On the left-hand side, we have a plain text recipe, which a sequence-to-sequence (encoder-decoder transformer) model parses to a semantically annotated one by augmenting the recipe with NER, RC, and coreference resolution notations. Then, a simple parser reads the annotated recipe and can print it as a workflow.

To support the development of methods for extracting workflows based on text, we introduced a new schema for annotating a collection of recipes with the above-described information. To facilitate the automatic extraction of such detailed information from recipes, we exploited transformer-based models (Vaswani et al., 2017) for the various information extraction tasks. Inspired by the work of Paolini et al. (2021), we cast information extraction as a structured prediction problem addressed using sequence-to-sequence modeling. However, we expanded it to jointly learn the tasks of NER, RC and coreference resolution. In addition, due to the length of recipe instructions—which tend to be longer than 512 tokens—we made use of the “long” transformer variants that can take longer text inputs. A visual depiction of our inference approach is provided in Figure 1.

Our contributions can be summarized as follows:

1. A new annotation schema, RiCoRecA, aimed at extracting workflows for IoT automation.
2. A dataset of 156 recipes labeled based on the RiCoRecA annotation schema.
3. A new transformer-based approach for generating workflows from recipes that is based on sequence-to-sequence modeling.
4. A comparison between two long transformer models for the joint tasks of NER, RC and coreference resolution.

**Figure 2:**
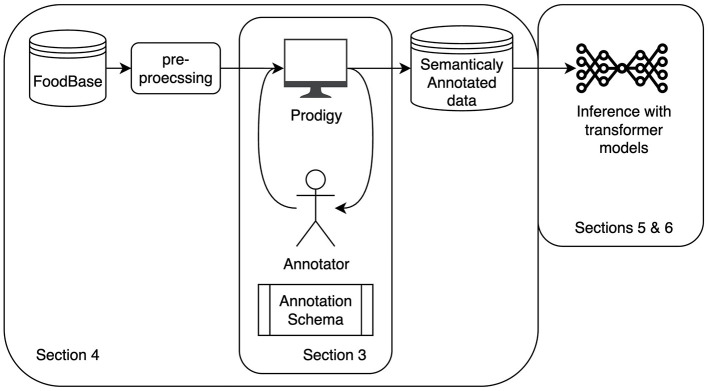
The figure shows a visual depiction of our overall methodology. It begins with the manually curated FoodBase dataset (Popovski et al., 2019) containing pre-labeled ingredients, following the initial pre-processing to clean the data and add NER labels using an off-the-shelf library. Then, a customized annotation interface [Prodigy (Montani and Honnibal 2017)] was employed for recipe labeling. The annotation process involved two iterative stages: an initial refinement of annotation guidelines followed by the final annotation phase. The resulting labeled recipes serve as the ground truth for inference using encoder-decoder transformer models. The figure indicates the sections where each part of the process is discussed.

Figure 2 provides a visual summary of our research methodology. The remainder of this paper is structured as follows. In Section 2, we review previous work on recipe workflow extraction using NLP and multi-modal approaches. Section 3 presents the details of the annotation schema for labeling named entities, the relationships between them, as well as coreferring expressions. In Section 4, we introduce our newly created dataset and the annotation methodology applied to develop it. We then describe our proposed approach to workflow extraction based on long transformer models in Section 5. This is followed by Section 6, which reports the results of evaluating our approach. Lastly, we provide a discussion of our results and future work in Section 7, and summarize our findings in Section 8.

## 2 Related studies

In this section, we present a number of studies that are more relevant to the problem of structured prediction from text more generally and the task of workflow extraction from recipes more specifically. We also review multi-modal approaches that have been proposed to address the latter.

### 2.1 Structured prediction

In the literature, a number of efforts have sought to transform text into a structured form such as abstract meaning representation (AMR) (Banarescu et al., 2013), the BabelNet meaning representation (Mart́ınez Lorenzo et al., 2022) or the resource description framework (RDF) graph format. For instance, Shirai and Kim (2022) utilized RDF triples to create a graph and solve a task such as the identification of ingredient substitutes. In our research, we parse text into an event structure. Within this framework, each cooking step is articulated as an individual event. Each event can be defined using the 5W1H criteria: “who,” “when,” “where,” “what,” “why” and “how” (Li et al., 2022).

Ventirozos et al. (2023) conducted a study that evaluated the semantic coherence of cooking recipe instructions in the context of orchestrating IoT devices. Notably, the semantic representation they employed bore similarities to that utilized in our research, primarily the 3W1H event representation, which also finds its roots in IoT control theory, which is used in our study. However, a key distinction lies in the fact that, in their approach, events were not interconnected, thus not facilitating the creation of (IoT) recipe workflows.

### 2.2 Recipe workflow extraction

Zhang et al. (2012) demonstrated how to employ a syntactic parser to extract procedural knowledge from texts (including cooking recipes). Focussed on process automation, they proposed that each step encompassed a set of slots to fill in, such as action verb, actee, instrument, post-condition and more. The hierarchical nature of a syntactic parse tree helps one to delineate the verb, which is on top of the tree, and by working their way down one could extract most of the lexical units. Our study does not rely on syntactic parsing; instead it utilizes a pre-trained language model (encoder-decoder transformer) to parse not only the named entities but also the entities used in each step and their relations. Specifically, one major difference in our annotation schema is that we link the various steps, utilizing relation extraction to model a control flow diagram.

A follow-up study that models the control flow was published a few years later by Schumacher (2015). They proposed a method for parsing procedural text (predominantly cooking recipes) into a workflow representation based on the Business Process Model and Notation (BPMN). By their definition, workflow components correspond to actions and products (i.e., ingredients and tools). In the same study, the notion of control flow extraction was also introduced. Four types of control were identified: (1) sequential, where actions follow a linear sequence; (2) disjunctive, where an action depends on a condition, e.g., “add sugar if you want”; (3) parallel, where actions can be executed in parallel, e.g., preheating the oven and laying the baking dish; and (4) repetitive, where actions need to be repeated until a certain requirement/condition is met, e.g., “repeat steps until all ingredients are mixed.” In their work, workflows were extracted by employing rule- and frame-based approaches utilizing the results of part-of-speech (POS) tagging, dependency parsing, and by exploiting the recipe's structure (e.g., paragraph indentation). In comparison, our work sought to utilize state-of-the-art transformers (Vaswani et al., 2017) to solve the structured prediction problem as a sequence-to-sequence modeling task.

More recently, Yamakata et al. (2020) annotated English cooking recipes with named entities and arcs (edges), representing relationships between the entities, based on a similar study on Japanese recipes (Mori et al., 2014). The authors defined ten named entity labels (e.g., food, tool duration, quantity, food state, action by food) and 13 edge labels. They automatically computed the flow graphs (similar to workflows) by first utilizing the encoder-only transformer BERT (Devlin et al., 2019) and then inferred the edges using a dependency parsing procedure. One main difference between their work and ours is that their generated workflows were not designed for IoT automation. Furthermore, our approach also learns actions that are dependent on some condition, similarly to Wu et al. (2022). In addition, we learn coreference resolution as part of the structured prediction problem, whereas previous work had focused only on NER and RC.

Rather than extracting workflows, Papadopoulos et al. (2022) investigated the extraction of programs from recipes. First, they constructed a taxonomy of actions and their lexical units (e.g., ingredient, tool, temperature, quantity). Then, using an encoder-decoder model, they parsed a recipe's text into a graph where each of the detected named entities is linked to the taxonomy, performing entity linking. They then connected each entity with relations to resemble a program. Again, our approach is different from theirs in that we learn coreference resolution (in addition to NER and RC) as well as actions that are contingent on certain conditions.

Other approaches involve predicting the workflow of a cooking recipe given the entities (Jermsurawong and Habash, 2015; Kiddon et al., 2015) or predicting just the named entities themselves (Wróblewska et al., 2022; Harashima and Hiramatsu, 2020; Gunamgari et al., 2014).

### 2.3 Multi-modal recipe workflow extraction

Recent studies have combined images with text or videos to extract richer information from cooking recipes. For instance, Zhang et al. (2021) utilized images to extract ingredient information not explicitly mentioned in the cooking steps. Meanwhile, Paulius et al. (2018) utilized annotated cooking videos to compose “task trees” (chains of events needed for a certain outcome) by fusing knowledge based on object similarity. Recent work also demonstrated how instructional videos can be automatically labeled by a model trained in an unsupervised manner (Piergiovanni et al., 2021). Our work is different from these in that we rely on text only, and leverage the knowledge learned by pre-trained transformer models.

## 3 Annotation schema

In this section, we introduce the annotation tags (labels) that were utilized in our study. According to our three NLP tasks, we have devised NER tags, RC tags and lastly an entity tracking scheme for coreference resolution. Table 2 presents our NER and RC tags and a short description of each.

**Table 2:** Summarized descriptions of our NER and RC tags.

**NER tags**	**Description**	**RC tags**	**Description**
Action	predicate, denoting a process	Modifier	Parameter to a process
Ingr	Ingredients or ingredient products	Member	Part of process
Tool	Tools, devices, equipment	Or	Code Statement “Or”
Part of *	Part of **Ingr** or **Tool**	Join	Denotes parallelism
Coreference of *	Reference to **Ingr** or **Tool**	Dependency	Process depended on another process
Msr & Sett	Measurement of **Ingr** and setting of **Tool**		
Why	Reasoning or justification for a process		
If	Code statement “If”		
Until	Code statement “Until”		
Repeat	Code statement “Repeat”		
State of *	Denotes condition of **Ingr** or **Tool**		

### 3.1 Named entity recognition tags

We delved into IoT control theory to create the appropriate named entity labels. Specifically, we drew inspiration from the work of Milis et al. (2019). They created a semantically enhanced IoT-enabled Intelligent Control System (SEMIoTICS). The control system involved a supervisor module facilitating the semantic modeling of IoT workflow composition and reconfiguration. The backbone of the system relies on a database of semantically annotated signals. Each signal is a quadruple, where each slot would be one of the defined “Where,” “What,” “Why” (Ws) and “How” (3W1H). For instance, in order for the supervisor to acquire a temperature sensor (thermometer) reading from an oven, it would have the following quadruple:

1. *Where* → Oven: represents the location and answers the question “Where?”
2. *What* → Temperature: represents the studied property of the feature of interest and answers the question “What?”
3. *Why* → State Measurement: represents the role of the signal or the parameter in the control system configuration and answers the question “Why?”
4. *How* → Celsius: the measurement unit of the property, where applicable, and answers the question “How?”

Our named entity labels were inspired by the 3W1H semantics and were adjusted to facilitate cooking recipes and, more generally, instructional text. Firstly, we wanted to include control flow extraction (see Section 2.2) in the design. Secondly, the annotation methodology (in iterations) described in Section 4.5 aided in refining the named entity types, relation types and the corresponding guidelines. For our study, we characterize the named entities as span-based; they can include one to multiple words. Below we present the named entity tags, as core and code statements, followed by the relation tags.

#### 3.1.1 Core

*Action* is usually a verb denoting the current process at hand. It pertains to the answer to the question “*What?”*, as in “What is happening in this step?” (e.g., collecting ingredients). Similar to the SEMIoTICS approach, the answer is typically a verb. For instance, in “Pre-heat the oven on 200 C” the verb “Pre-heat” denotes the type of signal (heating) that should be sent to an IoT device (an oven in this case).

*Entities* are categorized into expendables and durable, i.e., into ingredients and tools (i.e., devices).

- **Ingr**idients involve any ingredient or ingredient product.
- **Tool**s involve any concept of a tool. These could be appliances, cutlery, plates, worktops and even hands in some cases. In addition, tools tend to answer the question “*Where?”*. In our case, the location would refer to a part of the kitchen, which is usually an appliance or equipment. For instance, in “Pre-heat the oven on 200 C,” the word “oven” denotes the location that we want to pre-heat.

*Part of ingr and part of tool* were introduced to label the spans of words that refer to a specific part of an entity (e.g., “the icing of the cake,” “door of the oven”).

*Coreference of ingr and coreference of tool* are used for coreference between entities. This is further explained in Section 3.3.

*Msr and Sett* are two different labels, referring to the measurement and the setting. As one can see below, they both answer the question “How?”. The measurement, as the name suggests, refers to the quantity of an entity, usually an **Ingr** (e.g. “2 tbsp of sugar”). Setting refers to the setting of a **Tool** (e.g., “Pre-heat the oven on 200 C”). It's worth noting that in some cases, these can refer to verbose descriptions, e.g., “cover pan with water, cover everything plus one inch” (**Msr**) or “Reduce heat to a simmer” (**Sett**).

*Why* in the context of SEMIoTICS, this pertains to the reason why a signal is sent to an IoT device, e.g., to acquire a state measurement or to increase a value (e.g., heating). Moreover, this measurement is manually set by the engineer/technician or automatically by downloading the information from the Internet to fit the current plan's ontology. In our case, the “Why” refers to the span of words that explain the reason behind an action. For instance, a reason to pre-heat an oven could be “If you don't preheat your oven the temperature won't be hot enough and the end result may be a heavy, under-cooked mess.”

#### 3.1.2 Code statements

Code statement is a parent term that refers to coding keywords, such as “if,” “for” or “until.” These are crucial for the development of automation since they are the backbone of sensor-type devices. For instance, “bake until golden brown,” could refer to continuing with baking until (conditional statement) a camera-sensor would detect that the food entity is golden brown. Another example is “whisk until no lumps.” In our study, these keywords are:

*If tests* if a certain condition holds (e.g., “if,” “whether”) and determines if an alternative predicate or entity applies.

*Until* denotes doing an **Action** until a state is met (e.g., “until,” “while”).

*Repeat* signifies repeating an **Action** for a number of times (e.g., “for,” “repeat”).

*Stt* refers to the **state of ingredient** and **state of tool**. One can view them as programming conditions referring to a state. For instance, in the above example “bake until golden brown,” the underlined is a state of the ingredient linked to the **Until**.

### 3.2 Relation classification tags

We perform relation classification by considering the predicate, i.e., **Action** or a Code Statement (from **Until**, **If** and **Repeat**), as the root of a tree. The entities and the remaining lexical units are linked to the predicates. We defined the following relations:

**Modifier **is a type of relation that applies when a tagged word span is a direct argument, referring to another word span. Typically, these would be **Msr** or **Sett** referring to entities (e.g., “200 C” would refer to “oven”) or Stt_* referring to a Code Statement (“no lumps” would refer to “until”).

**Member **denotes which lexical units belong to which predicate. Every lexical unit needs to be connected; if the above *Modifier* does not apply, then Member would be used. In addition, it is used when a predicate is part of another predicate, such as between the predicates in Line 6 and Line 5 in Table 1.

**Or **is affiliated with code statements. It denotes whether one predicate or another should be used; the same applies to entities.

**Join **is used to denote when two predicates should happen in parallel.

**Dependency **is a type of relation that denotes which predicates are dependent on which. Once the annotator fills in the *Dependency* links, a timeline of predicates could be formed.

**Figure 3:**
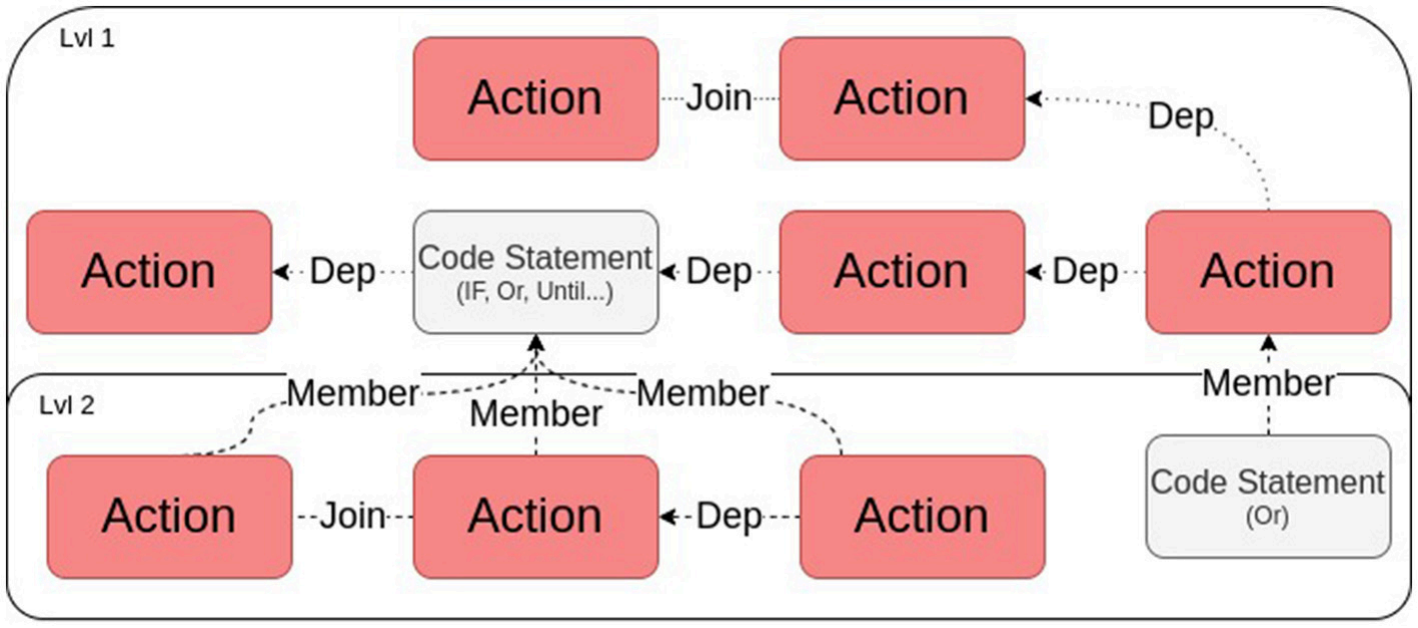
Demonstration of how predicates can be linked. The red boxes are **Actions** (actuations) and the gray ones are *code statements* (related to sensing). The “Dep” links stand for predicate Dependency. Lvl 2 shows nested predicates.

Figure 3 presents a high-level demonstration of how predicates can be connected using the aforementioned relationships.

### 3.3 Entity tracking

#### 3.3.1 Named entity recognition tags for coreference resolution

Cooking recipes use culinary jargon and are often written in colloquial language. Certain terms are expected to be known. For instance, the word “batter” would be anticipated within a pancake recipe. Fang et al. (2022) and our observations show that cooking recipes use four types of coreferring expressions/anaphors^2^ for ingredients. Primarily, we have two one-to-one relationships. These are: (1) referring to the exact same entity, without undergoing a process and changing its state, but with different wording (e.g., a pronoun). (2) Referring to the exact same entity, but being transformed through a process (e.g., “marinated meat”). We also consider two one-to-many relationships with the same distinction of with and without any state change. In our study, we grouped all the coreference types under one tag for **Ingr** and **Tool**, i.e., **Coreference of ingr** and **Coreference of tool**, respectively. As a general rule, any wording(s) that refer to a previous entity and is different should be considered Coreference of * instead (e.g., “dough” is a **Coreference of ingr** referring to water, yeast, flour etc., whereas “preheated oven” is a **Coreference of tool**). In some cases, it could even be the same word if and only if the latter entity has added entities onto it, despite preserving its name.

#### 3.3.2 Spreadsheet filling

The difficulty of processing instructional text is further compounded by the omission of certain descriptive parts that are implied. We noticed that certain actuations are considered to be based on common sense, and make the assumption that an agent (a human) should be able to track the entities. For instance, if one says “remove the chicken from the oven,” they imply switching off the oven's heating, and the oven would not be in the image depicting the next step. Hence, state tracking applies to the entities. As explained in Section 4.3, this was done with the aid of a spreadsheet, which allows for recording which entities are involved in each predicate, thus uncovering the entities that certain coreferring expressions pertain to.

More information on the annotation rules and processes can be found in the annotation guidelines^3^.

## 4 Dataset annotation

To test our hypothesis that an NLP model can perform NER, RC and coreference resolution to extract a workflow from a recipe, we needed to create a suitably annotated dataset for the purposes of developing models. Our target dataset was not intended to be large enough for training machine learning models from scratch. Consequently, we propose an annotation task that can be executed by even one person, with the expectation that a pre-trained transformer can achieve near-human-like inference. Below, we describe the annotation methodology and the dataset in detail.

### 4.1 Annotation methodology

The annotation process consists of two sections. Firstly, the annotator utilized a customized Prodigy^4^ interface to complete the NER and RC annotation tasks. After completion, the results were saved into a downloadable CSV file, where the annotator would need to fill in which initial entities are present in each predicate. In cases where the reference of an ingredient had changed (e.g., “flour” should be present where “dough” is), it was annotated with its original entity (e.g., “flour”); we refer to this task as coreference resolution. Also, this format allowed the annotator to omit any sentences or phrases that are comments, comments ensuring how a state should be without contributing to the process (e.g., “It should be piping hot right now”); and generally, comments that are typically not in the imperative form which do not contribute to the process. Additionally, duplicate actions were not considered.

### 4.2 Source dataset

We used the FoodBase corpus (Popovski et al., 2019) for our study. The recipes were extracted from allrecipes.com, one of the most popular cooking websites, accessible to everyone who wishes to contribute any recipes. Specifically, we used a manually curated dataset which was annotated with ingredient semantic types adhering to the taxonomy of the Hansard Corpus^5^. The ingredient taxonomy served two purposes in our study: 1. expediting the NER annotation process 2. supporting future studies on graph clustering (covered in Section 7.2). The dataset was pre-processed using tokenisation (e.g., 425F to 425 F) to allow fine-grained span labeling in the first annotation iterations. Following this, to expedite the annotation process, we utilized spaCy's part-of-speech (POS) tagger to mark ACTION (action) tokens. Thus, the ACTION and INGR (ingredient) labels, documented in Section 3.1, were automatically labeled by the spaCy tool and the dataset's provided labels. Nonetheless, the annotator could change them during the NE annotation step should they perceive that they are inaccurate. The FoodBase corpus' dinner and lunch recipes were used since they contained the most mentions of tools and kitchen devices. The code for pre-processing and the dataset are freely available in github.com/FilipposVentirozos/RicoReca.git and figshare.com, respectively.

### 4.3 Annotation interface

**Figure 4:**
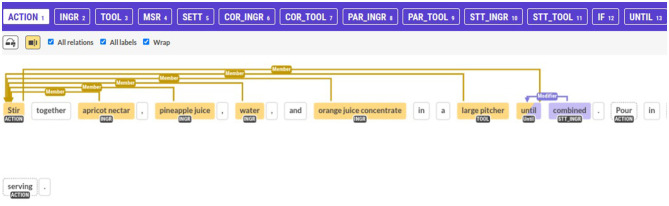
Part of an annotated small recipe with Named Entities and Relations. The Prodigy annotation tool by Montani and Honnibal (2017) was used.

An annotator would read a recipe and complete the following tasks: (1) named entity recognition (NER), (2) relation extraction and classification (RC), and (3) entity tracking for coreference resolution. Each annotator was assigned a username and a set of recipes to annotate via a web browser. Annotation was conducted remotely and asynchronously because Prodigy allows saving and resuming at any time. We commended this style of annotation, as a single recipe could take up to 30 min to annotate; in this way, annotators could work at their convenience and diligently allocate time to understand step dependencies, annotate, and evaluate their annotations. The landing page for each annotator was the Prodigy interface, a customized joint NER and RC annotation page. Figure 4 shows an example of the annotation view.

**Figure 5:**
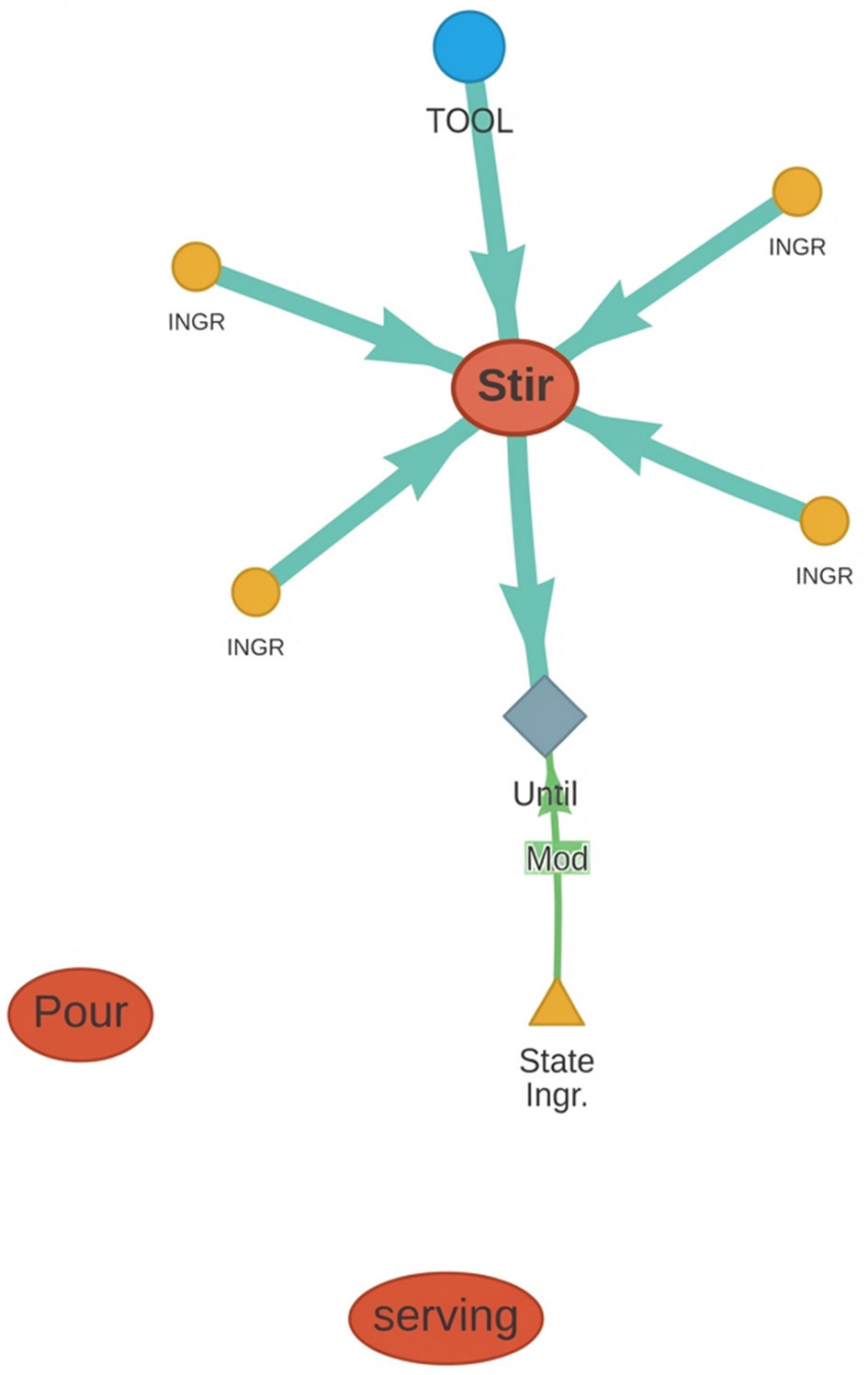
The graph interactive output from Figure 4. This section belonged under the previous output for the annotator's convenience.

This view includes an interactive graph that was generated using the Visjs library^6^ for the convenience of the annotator; an example is shown in Figure 5. In addition, a button was provided at the bottom of the page, and if pressed, it would download a spreadsheet (CSV file) auto-filled with the actions (predicates) and the entities that the user had annotated up to that point. Rows in this spreadsheet refer to the actions, and the columns correspond to all the entities. Annotators reviewed the spreadsheet using their preferred spreadsheet software (e.g., Excel) and filled in the cells with “1” where an entity is present in an actuation or left empty if not. The full annotation guidelines and guide to the graphical annotation interface are available as Supplementary material.

### 4.4 Annotators

Three annotators contributed to the annotation task, namely, the first three authors of this paper. These annotators represented diversity in terms of gender and geographic origins, hailing from Greece, Mexico and Saudi Arabia. While not native speakers of English, each annotator has demonstrated proficiency in the language, evidenced by postgraduate degrees in an English-speaking university. Additionally, they all have knowledge of NLP and linguistics. Lastly, they are moderate users of online cooking recipes and cooks.

### 4.5 Annotation phases

There were two phases in the annotation process. In the first one, the lead author was creating the guidelines and testing them with the second author. The process mimicked the DevOps methodology (Jabbari et al., 2016). The lead author was responsible for designing the guidelines, updating the annotation interface and ensuring that there were no issues in the annotation process. The second author then annotated based on the annotation scheme. The first author monitored their progress and received feedback on the annotation process, in order to plan the next iteration of guideline testing. In this strategy, fine-grained details were firstly annotated (e.g., specific units and metrics) followed by the annotation of coarse-grained ones, labeling everything under one tag. The decisions were based on the trade-off between the annotator's effort and the recipes' patterns (e.g., the span “fill with enough water to cover almost the top of the pot” is difficult to label as unit and metric, such as “two” → unit, “litres” → metric) relative to the semantic information gained. Once the guidelines were stable, the first phase was considered completed. For phase two, approximately 150 recipes were allocated to the first author and 50 to each of the second and third authors. The third annotator was given an opportunity to practice with a few trial recipes before annotating the set of recipes allocated to them.

### 4.6 Dataset statistics

The annotation process resulted in 156 labeled recipes. On average, each recipe contains 9.3 sentences and 133.4 tokens. Table 3 presents the frequencies of NER and RC tags in the labeled dataset. The duration of annotation varied with the recipe length: the shortest recipes, consisting of two sentences, required approximately 5 min; the longest one, consisting of 26 sentences, took an hour or more.

**Table 3:** The frequencies of NER and RC tags recorded by the first annotator.

**NER tags**	**Counts**			**RC tags**	**Counts**
	**Ingredient**	**Tool**	**Total**		
Action			2,290	Modifier	912
Entity	1,865	673		Member	4,762
Part of	295	50		Or	243
Coreference of	654	192		Join	146
Msr & Sett	305	312		Dependency	1,943
Why			85		
If			42		
Until			699		
Repeat			22		
State of	547	25			

The counts are based on spans labeled by the annotator. In total there are 8,056 NER tags and 8,006 RC tags.

Evidently, our dataset size is not big enough to train machine learning models from scratch. Our position is that we put forward an annotation task that can be done relatively easily and result in a dataset that can be used to fine-tune a pre-trained transformer.

### 4.7 Inter-annotator agreement

Cohen's Kappa is recognized as the metric of choice when dealing with inter-annotator agreement. It was developed to counter the percentage agreement between annotators, as it accounts for chance agreement, which percentage agreement (e.g., accuracy) does not (McHugh, 2012). However, studies in information extraction (Hripcsak and Rothschild, 2005; Deleger et al., 2012; Brandsen et al., 2020; Richie et al., 2022) have highlighted its inefficacy. Notably, in the context of NER, they mention that the task involves tagging sequences of words, which are not associated with the concept of true negatives found in typical classification tasks. These true negatives are necessary for calculating the Kappa statistic. Consequently, they concluded that the F1-score is more suitable. Furthermore, Cohen's Kappa is not suited for multi-label classification tasks, which are part of our information extraction task. Bearing all these points in mind, we opted for the F1-score. The bespoke F1-score metrics for all our information extraction tasks are described in Section 6.

In our study, 50 recipes were annotated by the second and third authors and evaluated against the first author. Table 4 displays the inter-annotator agreement results. Notably, both sets of agreements follow the same pattern. The highest agreement was observed in NER, followed by membership, then RC, and lastly, antecedents. Upon further inspection, we identified that the NER tags **Repeat**, **Part of Tool**, and the RC tag *Join* obtained the lowest agreement in both instances. These tags were also the most sparse in the dataset, especially in the more intricate recipes.

**Table 4:** The inter-annotator agreement between the first author vs. the second and third author.

	**2nd author**			**3rd author**		
**Type**	**F-score**	**Precision**	**Recall**	**F-score**	**Precision**	**Recall**
Antecedents	0.714	0.699	0.73	0.807	0.794	0.821
Membership	0.822	0.832	0.811	0.89	0.89	0.891
NER	0.886	0.874	0.898	0.908	0.916	0.9
RC	0.809	1.0	0.679	0.871	1.0	0.772

Section 6.1 mentions the types of evaluations that are presented in the Type column. Briefly, NER and RC refer to the tags in Table 2. However, the RC tag Member is an exception, as it falls under the Membership type, defined by the Member linkage tags and additionally by link traversal from other links. Lastly, the antecedent type pertains to coreference resolution.

## 5 Generative encoder-decoder transformer model

In the preceding sections, we detailed three types of annotations–NER, RC, and entity tracking–alongside the dataset. In this section, we first introduce the sequence-to-sequence task, explaining how a raw text recipe is parsed into an output that incorporates all three types of semantic annotations. We then discuss the transformer models employed in this study.

### 5.1 Sequence to sequence task

In the context of a sequence-to-sequence (seq2seq) encoder-decoder task, we formalize the parsing process of a given recipe *X* as follows: Given a recipe *X*, our objective is to transform it into an augmented output *Y*, which reproduces the recipe while integrating annotations that encapsulate the previously described semantic information. This can be mathematically represented by the function:


Y=f(X)

where *f* denotes the seq2seq model. This model maps the input sequence *X*—the raw recipe text—to the output sequence *Y*—the annotated recipe. The training of the model involves recognizing and encoding different types of semantic annotations such as NER, RC, and entity tracking, directly within the text of *Y*.

To incorporate the annotations into the format *Y*, we drew inspiration from the work described by Paolini et al. (2021). They used a T5 (Raffel et al., 2020) for structured prediction. They demonstrated and argued about different formats for doing NER, RC, and other NLP tasks. We adhered to their findings to: (1) generate tokens instead of numbers (e.g., it could be the predicate's count number instead), (2) repeat the entire input sentence in the output, and (3) augment the structured format for NER and RC by using the same special characters. Below, we show how we tangled all the information for one output. And following are some examples for reference.

For each token that is a predicate or a lexical unit, we parse as follows, assuming that token 1 is only of interest in this example:

tok_0 tok_1 ..., tok_n. → tok_0 [tok_1 | entities
| predicate | NER label | RC labels ] ..., tok_n.

As one can distinguish, we parse a token into a quintuple between the square brackets. In particular, we have the following spaces to fill in the quintuple, the:

1. token(s) themselves.
2. antecedents. This applies only to entities. In this space, would be listed the entities that it is composed of if any (e.g., the dough may be composed of flour, sugar, milk, yeast salt). If it is not a coreference but the actual entity, then it will have the same token as in space 1. Then, should the token not be an entity (e.g., predicate) then it would remain empty.
3. predicate it belongs to. This is straightforward for the lexical units. Nonetheless, the predicates also have this space filled. It's either themselves or the predicate they belong to. For instance, in the introductory example, the indented action “bake” (line 6) belongs to the “Until” (line 5).
4. NER label, these are described in Section 4 in more detail.
5. RC labels and the corresponding tokens, described previously in Section 4 in more detail.

The subsequent illustrations, which derive from Table 1, adhere to the format. Herein, they serve as references for elucidating the utilization of this format.

The “cut” found in line 2 in the right pane would be expressed as:

cut → [cut | | cut | ACTION | Dependency = Clean]

since it is a predicate, it does not have antecedents. It does not belong to another predicate, hence, the same word is repeated on the third space. Then, it is dependent on the previous predicate (i.e. Clean).

The “baking tray” found in line 7 is a TOOL type NER tag, and since it is firstly introduced there it does not have any antecedents, hence, it keeps its name. It belongs under the predicate “place.” Consequently, it would be written as:

baking tray → [baking tray | baking tray | place
| TOOL |]

The “bake” predicate found in line 9 is under the conditional statements “until” and “(For) 40–50 min” (regarded as a NER tag of Until). Hence, would be written as:

bake → [bake | | 40–50 min, until | ACTION |]

Lastly, the “potatoes” in line 12 would be written as:

potatoes → [potatoes | potatoes, olive oil, salt, pepper,
herbs, minced garlic | serve | INGR |]

Since the term potatoes refers not only to the potatoes used but also to the other added ingredients.

In Supplementary material, one can find examples of a whole recipe as input to output with this format. Moreover, it is worth mentioning that in our representation, we labeled spans as named entities, but without using the IOB or IOB2 format. Instead, all the tokens of a tag had the same label.

To be able to parse the annotations to the format mentioned above, further processing had to be done on the annotated data. Firstly, we extracted all the predicates from Prodigy and matched them with the spreadsheet ones. Then we had to link all the entities to the belonging predicate. However, a lot of them were not linked directly to it. Hence, we treated the problem as a graph, and by traversing the links recursively, we could link each entity to its predicate. In our calculations, when we traversed a recipe graph, we had to treat the *Dependency* and the *Modifier* relations as directional, whereas the rest as omnidirectional. Next, we filled in the entities that link to entities according to the algorithm described in Supplementary material. Lastly, we filled in the **Msr** and **Sett**, which did not specify the entity they were referring to, similarly to how we filled in the entities. The relevant code is provided in https://github.com/FilipposVentirozos/RiCoRecA.

### 5.2 Long transformers

We build on Paolini et al. (2021)'s work; hence, we opted for the same methodology of fine-tuning encoder-decoder models for our task. Nonetheless, it was evident that the outputs we targeted were quite lengthy, as an input to a model would be an entire recipe ranging from 2 to nearly 30 sentences, and the output would be an augmentation of that text, potentially 4–6 times longer. Consequently, a typical 512-token limit encoder-decoder transformer would omit valuable output. Therefore, we opted for transformers that can accommodate longer sequences. It is uncommon in the literature to find tasks involving short inputs and long outputs; however, the reverse is prevalent. A considerable amount of research has proposed various architectures for summarisation and parsing from longer to shorter text. Below, we document two recent long summarisation transformers that we employed in our experiments and can adapt to longer outputs.

#### 5.2.1 LongT5

The LongT5 (Guo et al., 2022) is characterized as a scalable T5 model (Raffel et al., 2020). Fundamentally, the T5 model adheres to the encoder-decoder architecture with trainable weights as delineated by Vaswani et al. (2017). Briefly, it comprises an encoder consisting of multiple blocks. These blocks refer to a grouping of neural network layers which are stacked one after the other. Characteristically, each block includes a self-attention layer (Vaswani et al., 2017), which utilizes trainable autoregressive weights to map onto the sequence itself, followed by a feed-forward neural network. The decoder is similar but incorporates a standard attention layer to map the trainable attention weights from the encoder's output. The LongT5 extends the T5's architecture by enhancing its attention capabilities for longer sequences. In Guo et al. (2022) two attention strategies were compared 1. transient global attention 2. local attention.

In our study, we utilized the transient global attention mechanism within LongT5, which modifies ETC's (Ainslie et al., 2020) global-local attention using a fixed block pattern. The mechanism divides the input sequence into blocks of *k* tokens, each block generating a global token by summing and normalizing the embeddings within it. This configuration allows each token to attend not only to nearby tokens but also to transient global tokens dynamically constructed during the attention phase. This approach minimizes additional parameters, requiring only T5-style relative position biases and layer normalization for global tokens. Such a structure enhances context comprehension and integration across longer texts, proving more effective in the summarisation tasks than the local attention.

#### 5.2.2 PEGASUS-X

PEGASUS-X (Phang et al., 2023) is an extension of the PEGASUS (Zhang et al., 2020). The PEGASUS followed a similar encoder-decoder architecture to T5 but was scoped for abstractive summarisation and was pre-trained with a self-supervised gap-sentence-generation objective (Zhang et al., 2020). PEGASUS-X, compared with its predecessor, accommodates an additional long input pre-training and uses staggered block-local attention with global tokens in the encoder.

Similarly to before, a block is a bucket which has a part of the text input. Again, we would separate the text input into *k* number of blocks. However, the staggered block-local attention method represents an advancement beyond LongT5's local and transient global attention. Specifically, staggering involves varying block boundaries across the various neural network layers in block-local attention, thereby enhancing the model's ability to incorporate global information. The use of staggered, overlapping blocks has proven to be a more effective strategy for long abstractive summarisation (Phang et al., 2023). However, the staggered blocks strategy is not the only difference between these models. For a comprehensive understanding of how PEGASUS-X diverges from LongT5, we refer readers to the study by Phang et al. (2023).

## 6 Experiments

Building on the preceding sections, this segment introduces our evaluation criteria–a bespoke approach tailored to our specific task. These criteria were employed to evaluate both the models and the inter-annotator agreement. Following this, we detail the parsing experiments conducted with the two models previously discussed, documenting their performance using the evaluation metrics we have introduced.

### 6.1 Evaluation criteria

The NER evaluation criterion was based on the SemEval F-score measure (Segura-Bedmar et al., 2013). Specifically, for NER we calculated for each token whether the token was:

1. *Correct*: classified correctly.
2. *Hypothesized*: A tag entry (i.e. the classified label of a span of word[s]) exists in the prediction but not in the ground truth.
3. *Missed*: A tag entry exists in the ground truth, but not in the prediction, the reverse from the above.
4. *Incorrect*: The tag entry between the prediction and the ground truth differs. For instance, in the ground truth, the tag entry expands into two tokens, whereas in the prediction, in one only.

Once we populated the above, we could record the precision and recall as follows:


$$\begin{array}{l} Precision=Correct/\left(Correct+Incorrect+Hypothesized\right) \end{array}$$ (1)

and the recall:


$$\begin{array}{l} Recall=Correct/\left(Correct+Incorrect+Missed\right) \end{array}$$ (2)

From these two, it was then easy to draw the known F-score formula.

The rest of the evaluations are rarely found in the literature. In relation extraction papers, there is always one class type per detected pair (Bassignana and Plank, 2022), resulting in a multi-class classification task. However, in our case, the membership, the entities and the RC tasks may contain from zero to multiple labels (i.e. multi-label classification task). Hence, for these, we treated the evaluation slightly differently but built upon the aforementioned NER SemEval evaluation. We devised this metric instead, which includes set theory:

1. *Correct*: the intersection (∩) between the ground truth items and the predicted.
2. *Hypothesized*: the difference (−) of predicted items minus the ground truth items.
3. *Missed*: the difference (−) of ground truth items minus the predicted items.

Following, the precision and recall were calculated identically with the Equations 1, 2 respectively, but by omitting the not available “Incorrect.” In conclusion, we have the following metrics, these follow the numbered points 2, 3, 4, and 5 from Section 5.1:

1. **Antecedents** are the prior entities to which entities are referring. It relates to the coreference resolution task. In the case that the entities are the main ones, introduced first, then themselves would classify as the correct answer. Non-entities were omitted in the calculation (e.g., predicates).
2. **Membership** the predicates that a lexical unit or predicate belongs to. It will always be populated.
3. **NER** the only task that we evaluated with the exact SemEval F-score measure.
4. **RC** of the remaining relationships (apart from membership) that a lexical unit or predicate may have. It can be none or multiple.

Lastly, after the output was generated, there had to be an alignment with the input text. The reason is that not all tokens are copied from the input into the output, and some may be altered. Therefore, in Paolini et al. (2021), the authors wrote a dynamic programming algorithm based on Needleman and Wunsch (1970) for token alignment. Instead, we incorporated^7^ the Myers (1986) dynamic programming algorithm, which is used in git diff.

### 6.2 Parsing using transformers

For our experiments, we utilized an A100 80Gb Ampere NVIDIA GPU. We compared the two aforementioned models, PEGASUS-X^8^ and LongT5^9^ using their large provided models from the HuggingFace library (Wolf et al., 2020).

Chiefly, we opted for a conservative learning rate of 5*e*−4 and a maximum of 20 epochs since the dataset did not have many instances. Due to the long output of our data, we had an input sequence length of 420 and an output of 2,580 tokens to cover even our most lengthy recipes. However, due to the large model sizes and the long token sequences, we could use, at most, a batch size of 2 and 1 for the PEGASUS-X and the LongT5, respectively. We opted for five-fold cross-validation trained on the 156 first author's annotated recipes to perform our experiments. The best-performing epoch model was selected based on the Rouge1 score (Lin, 2004). The models' results are averaged and depicted in Table 5. The outcomes derived from the five-fold cross-validation demonstrated minimal divergence among the distinct folds. The table results clearly indicate the superior performance of the PEGASUS-X transformer compared to the LongT5 across all evaluative metrics. Furthermore, a detailed analysis of the inference datasets revealed a tendency for the LongT5 model to erroneously produce repetitive token sequences within specific recipe outputs. Lastly, similarly to the annotators (Table 4), one can distinguish that the NER was again leading the scoreboard, followed this time by the RC and then the Memberships and the antecedents.

**Table 5:** The results of the PEGASUS-X and LongT5 transformers.

	**PEGASUS-X**			**LongT5**		
**Type**	**F-score**	**Precision**	**Recall**	**F-score**	**Precision**	**Recall**
Antecedents	0.626	0.666	0.591	0.228	0.274	0.196
Membership	0.835	0.841	0.829	0.38	0.386	0.374
NER	0.883	0.894	0.873	0.549	0.695	0.457
RC	0.83	1.0	0.709	0.462	1.0	0.302

They were fine-tuned, every time, on a five-fold cross-validation on the first author's annotators. The scores shown are the averages.

Once the parsing was generated, we could visualize a graph from it, similarly to Figure 5. However, not all predicates were connected, since some relations were missing. This is evident from the low recall the RC had, view Table 5.

## 7 Discussion and future work

### 7.1 Discussion

From the experiments, one can notice that the least challenging task is the NER. We believe there are two factors contributing to that. Firstly, the NER was framed as a multi-class, whereas the rest were multi-label, also having the no-label option. The latter is a considerably more difficult task since there are more degrees of freedom. Secondly, for the transformers, we believe that the type of word is relatively easy for them to classify without investing heavily in fine-tuning due to the distributed semantic knowledge they have obtained through pre-training. Moreover, the predictions of the transformers follow a similar trend to the annotators. From the NER and RC, the lowest scoring labels are again the NER tags **Repeat**, **Part of tool** and the RC tag *Join*. A fact that is exacerbated by the low volume of training data for these cases.

Identifying the antecedents from the labeled entities is admittedly perceived as the most challenging task, and the task scored lowest among the annotators. Upon annotating, we realized that it was not an easy task since a product could refer to 20 of the aforementioned ingredients. The task of tracking all the various ingredients was mundane and open to interpretation. Then, PEGASUS-X had a significant loss in predicting the antecedents compared to the rest of the tasks. Upon closer inspection, it was evident that some errors occurred during generation (e.g., repeated entities or introduced non-existent ones). Nonetheless, most errors resulted from the lack of reasoning and the word giveaway. Specifically, we noticed four themes:

1. Typically, at the start of a recipe, an ingredient is introduced with its full name. Then, further below the recipe, the ingredient is congested to one word (e.g., halibut filets → filets). This trait sometimes confused the transformer to not label the whole span (e.g., “halibut fillets” in this case).
2. In a similar fashion, main ingredients which undergo a process and other ingredients are added to them but are referred to with the same or part of the first main ingredient, have their added ingredients neglected.
3. On the contrary, if there was a word indicating part of an entity but had different wording (e.g., “inside [the turkey]”), PEGASUS-X could instead refer to other aforementioned entities rather than its entity (“turkey” in this case).
4. Lastly, there was a difference in interpretation. Should an entity be introduced in the latter stage of a recipe, PEGASUS-X assumed in some cases that it was a coreference instead.

We recognize that these errors are due to the missing knowledge of cooking and the lack of reasoning. The annotators could identify the cooking jargon and make a better judgement when entities are present.

Moreover, in certain instances, the coreference algorithm, explicated in Supplementary material, could not accurately attribute all the entities to their corresponding referents. The trait was evident when a predicate introduced multiple new coreference wordings. Since there was no one-to-one mapping (we only knew which entities were present), the algorithm proved ineffectual in correctly assigning all referential associations. Nevertheless, such occurrences were notably infrequent.

In the literature, akin to our entity tracking methodology, a growing interest has emerged regarding detecting entities throughout a process (Dalvi et al., 2019), including imagery (Zhang et al., 2022). Nevertheless, we identified a lacuna in the literature of additionally inferring their coreferences. Unquestionably, for automation, the knowledge of entity tracking is essential; however, coreference resolution could have the added benefit of retrieving the semantic meaning for each co-referred entity when interacting with the user. A potential resolution could have been to let the annotators assign links in the Annotation GUI between the co-referred ingredients. Despite its merits, this strategy would significantly obfuscate the annotation process and would not allow the further utilization of the dataset to detect entities when not mentioned, since this strategy tracks only the entities that are viewed and not the omitted ones.

Another challenging task that PEGASUS-X faced was to identify long-range relationships. It was noticeable with long *Dependency* RC links. If a predicate depended on one, many predicates ago (e.g., the first sentence says to “preheat” the oven, and the last one says to “bake”), it was challenging to identify. PEGASUS-X omitted most of these relationships; hence, the low recall in Table 5. Similarly, the annotators had low recall values (Table 4) since they often disagreed or were confused about long-range dependencies. It may be that these links require opting for non-machine learning solutions, such as inductive approaches (Nieves et al., 2020).

From the results in Table 5, one can distinguish the gap between PEGASUS-X and LongT5. If one averages the scores across all four tasks, it shows a difference of 39% F-Score. Then, if one views Tables 4, 5, one can recognize that the difference in scoring between PEGASUS-X and the annotators is subtle. The superiority of PEGASUS-X over LongT5 can be attributed to its architectural advantage of utilizing staggered blocks. Our results coincide with PEGASUS-X performing better in abstractive summarisation (Phang et al., 2023); however, we cannot confirm what feature or features make it superior. We are inclined to believe that the architecture is the reason since, during their training, both could reach peak performance on the validation set. This begs the question of what architecture, pre-training data, and task is best for structured prediction using transformers (Paolini et al., 2021). Although there has been an investigation (Wang et al., 2022) on pre-training for structural prediction, we believe there is more room for exploration for joint structured prediction on long transformers. Our view is compounded by the fact that different architectures for long sequences performed better than the shorter ones; this was evident in shorter and longer abstractive summarisations (Phang et al., 2023). Another element for investigation is the output format. He and Choi (2023) demonstrated that opting for a more verbose approach, an entirely composed natural language instead of using special tokens (i.e. ‘]', ‘=', ‘]'), proved a better strategy. Nevertheless, it remains a challenge since our dataset has long inputs and exceptionally long outputs, making this option prohibitively expensive for our infrastructure.

### 7.2 Future work

A primary obstacle before parsing the generated workflow for device automation is the alignment of predicates to atomic services. By atomic services, we define the lowest level actuation and sensor services that are included in a device. In cooking, many processes are described succinctly, which may not be adequate information for an IoT or IoRT (e.g., “bake” instead of “set temperature on, open door, insert dish, ...”). Admittedly, there have been studies to augment the details of cooking predicates. For instance, Donatelli et al. (2021) looked at the problem of aligning recipe actions across recipes to retrieve more elaborate actions. We leave this task for future work.

Further investigation is needed to capture the entities that are present in a predicate but are not mentioned, most often because it is expected from the reader to make the inference. These could be added to the output sequence. An additional investigation needs to analyse the possible formats and entity checking.

We hope that this work can challenge future investigations in this area. There are multiple directions for research. Foremost, transformers trained on large corpora of cooking datasets with this methodology can harness the knowledge to achieve several downstream tasks. They could suggest substitutes to entities (i.e. ingredients, tools) but also to measurements and device settings depending on the context. Moreover, transformers with the language modeling setting can generate the most probable tokens based on the input in an autoregressive manner. We believe that using a similar setting could aid in predicting the next cooking step, including variables related to device operability.

We propose a more extensive investigation in the language modeling domain of NLP to explore alternative models for executing this task and evaluating the results. Decoder-only architectures such as GPT-5 (OpenAI et al., 2024), Claude (Anthropic, 2025) or Gemini (Gemini Team et al., 2025), have gained prominence in NLP owing to their straightforward training approach, which involves predicting the next token in an autoregressive manner. While this approach may yield promising results for our task, significant further experimentation remains necessary. Given the highly context-dependent nature of the problem, a long-context decoder model would be essential for in-context learning (Dong et al., 2024). However, such models often struggle to effectively capture long-range dependencies–a limitation consistently observed in relevant benchmarks (Li et al., 2024).

Lastly, we hope to raise awareness of sibling domains in which textual manuals or process documentation can serve as specifications for programming IoT-enabled systems. Beyond recipes, domains such as automotive maintenance, DIY home improvement, electronics assembly and testing, agricultural equipment operation, laboratory protocols, building and HVAC commissioning, smart appliance installation, network device configuration (e.g., routers and cameras), clinical device setup and calibration, industrial preventive maintenance, and drone/3D printer/CNC workflows can benefit from machine-assisted interpretation of procedures to guide users and automate routine steps. By transforming free-text instructions into executable task graphs aligned with device APIs and validated via sensor feedback, such systems can improve safety and compliance, reduce error rates and training time, increase reproducibility and auditability, and broaden access for non-expert users across heterogeneous devices and settings.

## 8 Conclusion

The current study presented a novel annotation schema. For its development, three annotation tasks were devised. Three annotators performed these tasks: NER, RC and entity tracking. The annotation campaign involved multiple iterations for optimizing the annotation guidelines, and it sought to adhere to IoT control system design principles. After the annotation task was completed, a codebase parsed the individual annotations into a unified representation. This included coreference resolution derived from entity tracking. The format was inspired by Paolini et al. (2021) and was designed to be compatible with encoder-decoder transformers, enabling both training on the processed examples and inference from textual recipes. We opted for “long” transformers, the PEGASUS-X and LongT5, since the recipes and their output format were too long for conventional encoder-decoder transformers. PEGASUS-X performed better than LongT5. However, both of them failed to identify some entities' coreference and could not uncover certain relations.

## Data Availability

The original contributions presented in the study are included in the article/Supplementary material, further inquiries can be directed to the corresponding author.

## Footnotes

1 Both IoT and IoRT devices are within the scope of our study, although we will use “IoT” to broadly refer to the two types of devices throughout this paper.

2 Anaphora resolution bears similarities with coreference resolution in that it focusses on identifying anaphors in a text and their corresponding antecedents. Anaphors are words or phrases that refer to earlier-mentioned words or phrases (antecedents).

3
https://figshare.com/articles/online_resource/RiCoRecA_i_Ri_i_ch_i_Co_i_oking_i_Rec_i_ipe_i_A_i_nnotation_Schema_Annotation_Interface_Guidelines_/25853902

4 Prodigy (Montani and Honnibal, 2017) is an annotation tool by the creators of the NLP toolkit spaCy (Honnibal et al., 2019).

5 The Hansard semantic tags are part of the Hansard Corpus project(Davies, 2015).

6
https://visjs.github.io/vis-network/docs/network/

7 We utilized the https://github.com/explosion/tokenizations library, which is affiliated with the Honnibal et al. (2019) library.

8
https://huggingface.co/google/pegasus-x-large

9
https://huggingface.co/google/long-t5-tglobal-large
