# Supplementary material for: RiCoRecA: rich cooking recipe annotation schema
Source: Front Artif Intell. 2026 Jan 12;8:1550604. doi: 10.3389/frai.2025.1550604 (PMC12833278; doi:10.3389/frai.2025.1550604)
Supplement: Supplementary file 1 [file Supplementary_file_1.pdf]

Supplementary Material

1 SUPPLEMENTARY DATA/APPENDIX

1.1 Coreference Resolution from Annotations

Once annotated a recipe, we would obtain the NER tags of the entities in their prime form, i.e. **Ingr** and **Tool** and in their processed/different form, i.e. **Part of Ingr**, **Part of Tool**, **Coreference of Ingr**, **Coreference of Tool**. If these terms are not familiar, we urge the reader to view Section 3.1. Then we had knowledge from the spreadsheet of which entities were present for each actuation. We devised an algorithm to link each **Part of *** and **Coreference of *** to the initial entities, the (INGR, TOOL) antecedents. We briefly describe it here.

For each entity that had to be matched to its antecedents, we initially checked whether the linked predicates contained the same entity. If that was the case, all the retrieved antecedents for that entity were carried from the previous predicate. Should the antecedents not have been filled, we would view the linked predicate row from the spreadsheet to link with the indicated antecedents. Should that fail, we would traverse the graph to identify other entities linked to it. Since in our annotation guidelines, we had the "rule of hierarchy". The rule dictated that an entity label ought to be a *Member* of another entity if they refer to the same antecedents under the same predicate. For instance, the **Part of tool** "lid" would be linked as a *Member* of the entity **pot**, since the lid is part of the pot. Thus, by traversing via the "rule of hierarchy", we could fill in the antecedents for some entities. Next, should the above fail, we checked whether the coreference is a pronoun. Should that be the case, we filled in the antecedents referred to the previously linked predicates. The relevant code is available in github.com/FilipposVentirozos/RiCoRecA.

1.2 Dataset Input & Output Examples

This section demonstrates some examples of the input and output of our transformer model for reference. The output below is split into multiple lines to fit the boxes' width and improve readability; realistically, it would all be on one line.

2 SUPPLEMENTARY TABLES AND FIGURES

2.1 Figures

Example 1

Input

Combine kefir, blueberries, banana, almond butter, and honey in a blender. Process until smooth.

Output

```
[Combine |  | Combine | ACTION | ]
[kefir | kefir | Combine | INGR | ],
[blueberries | blueberries | Combine | INGR | ],
[banana | banana | Combine | INGR | ],
[almond butter | almond butter | Combine | INGR | ], and
[honey | honey | Combine | INGR | ] in a
[blender | blender | Combine | TOOL | ].
[Process |  | until | ACTION | ]
[until |  | until | UNTIL |  Dependency = Combine]
[smooth | kefir, blueberries, honey, almond butter,
banana | until | STT_INGR | Modifier = until].
```

Example 2

Input

Preheat oven to 350 degrees F (175 C). Place bacon and ground beef in a large, deep skillet. Cook over medium high heat until evenly brown. Drain and set aside. In a four quart casserole dish, mix together the bacon, ground beef, pork and beans, kidney beans, butter beans, lima beans, ketchup, onion, vinegar, dry mustard and celery. Cover and bake for 1 hour.

Output

```
[Preheat |  | Preheat | ACTION | ] [oven | oven | Preheat | TOOL | ]
to [350 degrees F (175 C) |  | Preheat | SETT | Modifier = oven].
[Place |  | Place | ACTION | ]
[bacon | bacon | Place | INGR | ] and
[ground beef | ground beef | Place | INGR | ] in a
[large, deep skillet | large, deep skillet | Place | TOOL | ].
[Cook |  | until | ACTION | ] over
[medium high heat |  | Cook | SETT | Modifier = stove]
[until |  | until | UNTIL |  Dependency = Place]
[evenly brown | ground beef, bacon | until | STT_INGR | ].
[Drain |  | Drain | ACTION |  Dependency = until] and
[set |  | set | ACTION |  Dependency = Drain]
[aside | aside | set | TOOL | ].
In a [four quart casserole dish | four quart casserole dish |
mix | TOOL | ],
[mix |  | mix | ACTION |  Dependency = set] together the
[bacon | bacon | mix | INGR | ],
[ground beef | ground beef | mix | INGR | ],
[pork | pork | mix | INGR | ] and [beans | beans | mix | INGR | ],
[kidney beans | beans | mix | INGR | ],
[butter beans | beans | mix | INGR | ],
[lima beans | beans | mix | INGR | ],
[ketchup | ketchup | mix | INGR | ], [onion | onion | mix | INGR | ],
[vinegar | vinegar | mix | INGR | ],
[dry mustard | dry mustard | mix | INGR | ] and
[celery | celery | mix | INGR | ].
[Cover |  | Cover | ACTION |  Dependency = mix] and
[bake |  | for 1 hour | ACTION | ]
[for 1 hour |  | for 1 hour | UNTIL |  Dependency = Cover,
Dependency = Preheat].
```
